# Belief Shift or Only Facilitation: How Semantic Expectancy Affects Processing of Speech Degraded by Background Noise

**DOI:** 10.3389/fpsyg.2018.00116

**Published:** 2018-02-08

**Authors:** Katherine M. Simeon, Klinton Bicknell, Tina M. Grieco-Calub

**Affiliations:** ^1^The Roxelyn and Richard Pepper Department of Communication Sciences and Disorders, Northwestern University, Evanston, IL, United States; ^2^Department of Linguistics, Northwestern University, Evanston, IL, United States; ^3^Hugh Knowles Hearing Center, Northwestern University, Evanston, IL, United States

**Keywords:** semantic expectancy, ideal observer, lexical processing, speech perception, background noise

## Abstract

Individuals use semantic expectancy – applying conceptual and linguistic knowledge to speech input – to improve the accuracy and speed of language comprehension. This study tested how adults use semantic expectancy in quiet and in the presence of speech-shaped broadband noise at -7 and -12 dB signal-to-noise ratio. Twenty-four adults (22.1 ± 3.6 years, mean ±*SD*) were tested on a four-alternative-forced-choice task whereby they listened to sentences and were instructed to select an image matching the sentence-final word. The semantic expectancy of the sentences was unrelated to (neutral), congruent with, or conflicting with the acoustic target. Congruent expectancy improved accuracy and conflicting expectancy decreased accuracy relative to neutral, consistent with a theory where expectancy shifts beliefs toward likely words and away from unlikely words. Additionally, there were no significant interactions of expectancy and noise level when analyzed in log-odds, supporting the predictions of ideal observer models of speech perception.

## Introduction

Everyday conversations require listeners to rapidly comprehend language as speech input unfolds over time. To do this, individuals must match the spectrotemporal aspects of the speech input to lexical representations stored in long-term memory. This matching process is facilitated by *semantic expectancy*, which is the ability to apply general conceptual and linguistic knowledge to incoming language input (e.g., speech or text) in order to facilitate language comprehension (Rönnberg et al., 2011).

As individuals process language incrementally, they continuously build up contextual representations and use that context to build up semantic expectancy to help them identify subsequent words (Repp, 1982; Kamide, 2008; Rigler et al., 2015). This strategy can be particularly helpful for processing language when the speech input is degraded (Rönnberg et al., 2013; Winn, 2016). For instance, Winn (2016) utilized pupillometry to show that individuals exert less cognitive effort when processing noise-band vocoded sentences with high predictability than noise-band vocoded sentences with low predictability. Specifically, noise-band vocoding obscures the word form by restricting the spectral fidelity of the speech input. Semantic expectancy overcomes this reduced spectral fidelity by constraining the set of likely word candidates based on the preceding linguistic input and ultimately making it easier for the listener to identify the degraded word form. These results have implications for our daily lives. For example, most conversations occur in environments that contain competing sounds, such as other talkers and environmental noise, that degrade the target speech input. Thus, individuals rely on semantic expectancy in these environments to help sustain their online speech processing and language comprehension.

Decades of research have advanced our understanding of the effects of semantic expectancy on speech identification. For example, studies that have varied the amount of contextual information (low, medium, or high) in a sentence have shown increasing benefit for speech recognition as the level of contextual information increases (Benichov et al., 2012; Lash et al., 2013). We also know that semantic expectancy aids identification of a word in less natural tasks. For instance, lexical decision tasks, in which participants must decide whether a target word is a real word or a non-word, demonstrate benefits in accuracy and speed of response when there is expectancy information in the sentence carrier preceding the target (Aydelott et al., 2006; Goy et al., 2013). Finally, semantic expectancy is known to change the proportion of identification responses more for stimuli that are acoustically less clear (Connine, 1987; Borsky et al., 1998). For example, Borsky et al. (1998; cf. Miller et al., 1984; Connine, 1987) presented listeners with sentences like (A) where the target word was a token on an acoustic continuum from “coat” to “goat.”

(A) The elderly grandma stopped to button the [COAT/GOAT] in the corner closet.

They showed that the semantic expectancy had the biggest effects on proportion of responses for tokens in the intermediate, ambiguous part of the continuum.

It is still an open question, however, *how* semantic expectancy improves identification. One proposal, predicted by optimal integration or ideal observer models of speech perception (Clayards et al., 2008; Norris and McQueen, 2008; Bicknell et al., 2016; Bicknell et al., unpublished) among others, is that semantic expectancy improves identification by shifting listener beliefs toward likely or predicted words and thus away from unlikely ones. That is, if a word embedded in noise is preceded by a context setting up a semantic expectancy, that expectancy will make listeners more likely to identify the word as something semantically likely and thus less likely to identify it as something semantically less likely. Under this theory, the expectancy would improve accuracy relative to a neutral baseline if the word in noise was a semantically likely word, but decrease accuracy relative to a neutral baseline if the word in noise was a semantically unlikely word. We refer to this theory as *belief shift*.

An alternative possibility is that semantic expectancy facilitates the processing of semantically likely words, but does not bear any negative effects for a target word that was neutral or not semantically related. Such a pattern has previously been reported in analyses of duration measures, such as the time to name or categorize a word. For example, Duffy et al. (1989) compared the speed to name a target word based on preceding expectancy information in reading. Results demonstrated faster naming times for congruent expectancy (B) but no significant difference in naming latencies when comparing incongruent expectancy (C) to a neutral baseline (D), showing no penalty when the target word was preceded by incongruent expectancy information.

(B) *Congruent:* The barber trimmed the mustache.
(C) *Incongruent:* The barber trimmed the artifacts.
(D) *Subject–verb neutral:* The woman saw the mustache.

The priming literature also supports this possibility. For instance, presenting semantically related primes prior to the presentation of the target signal speeds target identification (Ferreira and Griffin, 2003; Sheldon et al., 2008; Obleser and Kotz, 2009). However, there are no detrimental effects if the prime is unrelated.

These results all suggest a mechanism in which semantic expectancy can *facilitate* the processing of a related target, but yields no negative effects if the target word is unrelated. We refer to this theory as *facilitation-only*. Studies that support this theory show that congruent semantic expectancy decreases response time and shortens latency, but that conflicting semantic expectancy does not change response time compared to a neutral baseline. However, what has been unexplored is whether these results for reaction time (RT) transfer to task accuracy. Specifically, this theory would predict that semantic expectancy would improve accuracy relative to a neutral baseline for a semantically likely target word, but not predict that semantic expectancy would decrease accuracy relative to a neutral baseline if the target word is not semantically likely.

The results of nearly all of the studies that have been performed on semantic expectancy in speech perception are consistent with either of these theories. The reason for this is that the majority of studies that have been performed have shown that a target word could be identified more accurately when it was predictable (i.e., in when *congruent* expectancy information is present), relative to either a *neutral* expectancy or when one of its competitors was highly predictable and it was unpredictable (i.e., when *conflicting* expectancy information is present). Both of these theories would predict a difference between *congruent* and *neutral* expectancy, because both predict congruent expectancy improves identification. Both of them would also predict a difference between *congruent* and *conflicting* expectancy, although for different reasons. In the belief shift theory, the difference between congruent and conflicting would arise both because the congruent expectancy shifts beliefs toward the target relative to neutral and because the conflicting expectancy shifts beliefs away from the target relative to neutral. In the facilitation-only theory, however, this difference would arise solely because the congruent condition is predicted to improve accuracy above a neutral baseline, while the conflicting condition would result in equivalent accuracy to that baseline. The critical question to distinguish these theories, then, is whether conflicting expectancy results in accuracy below a neutral baseline.

To our knowledge, only one paper has reported an experiment that included both conflicting expectancy and a neutral baseline in a spoken word identification task (Miller et al., 1984, Expt. 1). However, this study did not analyze the conflicting vs. neutral contrast directly. Nevertheless, the data patterns visualized in its figures are suggestive of a difference between neutral and conflicting expectancy. Additionally, this was a voice-onset time (VOT) continuum study; therefore, whether the semantic expectancy matched a given token was not clearly defined for tokens near the category boundary. The primary goal of the present study, then, is to perform a more rigorous test distinguishing these hypotheses.

Specifically, we present results from an experiment on the perception of speech in noise comparing all three conditions of semantic expectancy: congruent, neutral, and conflicting. The study employed a four-alternative-forced-choice behavioral paradigm to evaluate listeners’ identification of sentence-final words in each of the three different expectancy conditions. Our expectancy conditions vary to make the final word of the sentence semantically *congruent* with the expectancy information in the sentence carrier, semantically *conflicting* with the expectancy information in the sentence carrier, or contain no semantic information that supports a particular final word (explained further in the section “Materials and Methods”).

Using a speech in noise paradigm yields a range of advantages. For one, it allows for an unambiguous definition of the congruent and conflicting conditions, since the identity of the word embedded in the noise is not debatable (whereas when using an acoustic continuum such as a VOT manipulation, the actual identity of the word is arguably ambiguous for intermediate VOT values). Additionally, this design allows for the use of a wide range of target word pairs that are distinguished by various different phonetic cues, instead of using a single target word pair as in Miller et al. (1984), which limits the ability for listeners to use unnatural strategies to complete the task. Finally, this design allows for comparing multiple noise levels, including quiet, to determine whether the influence of semantic expectancy changes with noise level.

This final aspect of the design is also motivated by a secondary goal of this study. While it is well known (as described above) that semantic expectancy has the largest effect on identification for acoustic tokens that are especially ambiguous for a listener when identification is measured in proportions, optimal integration or ideal observer models of speech perception predict that the size of the effect of semantic expectancy is constant across acoustic tokens, when identification is measured on a log-odds scale (Bicknell et al., 2016; Bicknell et al., unpublished). This account is consistent with prior findings because a constant-sized effect on a log-odds scale will be largest in proportion around proportion 0.5 (i.e., when the stimulus is especially ambiguous), and will become smaller in proportion space closer to proportions 0 and 1. However, in addition to recovering this well-known qualitative pattern, this theory predicts a very specific quantitative shape of how much smaller the effect should be at any point. Conveniently, logistic regression analyzes data in log-odds, and thus this quantitative prediction is easily testable. Prior work has supported these predictions using data from acoustic continuum experiments (Bicknell et al., 2016; Bicknell et al., unpublished), and the secondary goal of this study is to test this prediction of such models on an experiment of speech identification in noise.

The predictions of each theory of semantic expectancy for accuracy in this experiment are as follows. Both the belief shift and facilitation-only theory predict that accuracy will be increased in the congruent condition relative to neutral. However, only the belief shift theory predicts that accuracy will be decreased in the conflicting condition relative to neutral. The secondary goal is testing the prediction of ideal observer models of speech perception that the effects of semantic expectancy are a constant size across acoustic conditions when measured on a log-odds scale. To test this prediction, we analyze the accuracy using (mixed-effects) logistic regression, which operates on a log-odds scale. In such an analysis, this predicts that expectancy condition will additively combine with noise level, i.e., that there will be no interaction.

## Materials and Methods

### Participants

Twenty-four native English-speaking adults (mean = 22.09 years, range = 18.8–31.04 years) were recruited from the Northwestern University community and greater Chicago area. Participants reported normal hearing and no prior history of speech and language services. Participants demonstrated typical cognitive functioning by scoring within two standard deviations of the mean on assessments in the NIH Toolbox (McDonald, 2014).

Participants completed an informed consent process prior to participation and were compensated for their time. All procedures were approved by the Institutional Review Board of Northwestern University.

### Stimuli

A total of 60 sentence carriers were developed for the present study. Forty sentence carriers had high semantic expectancy and 20 sentence carriers had minimal semantic information. Sentences were developed according to the criteria set forth by Bloom and Fischler (1980), including

(1) Each sentence carrier was made into a grammatically acceptable sentence by the addition of a single word.
(2) Each sentence carrier was six to eight words in length.
(3) Obvious clichés (e.g., overused expressions such as, “only time will tell” or phrases that are not meant to be taken literally such as “it is raining cats and dogs”) were avoided.
(4) A range of syntactic structures was included.

In addition to the sentence carriers, there were 20 word pairs (for a total of 40 words). Each word was monosyllabic and imageable. Words in each pair differed only in the initial phoneme and were distinct in either their place of articulation (*n* = 7 pairs), voicing (*n* = 5 pairs), or both place of articulation and voicing (*n* = 8 pairs). Each pair of words was matched with two sentence carriers with high expectancy and one sentence carrier with minimal semantic information. For the first high expectancy carrier, one word matched its semantic expectations based on criteria from Bloom and Fischler (1980) and Block and Baldwin (2010), and the other word did not (see examples below), and vice versa for the second high expectancy carrier. See example pair of two target words with three contexts in (1) and (2).

Examples of expectancy conditions:

(1) Target word: “bat”
  a. *Congruent expectancy*: The boy hits the ball with the **bat**.
  b. *Conflicting expectancy*: The mice are afraid of the **bat**.
  c. *Neutral*: The boy sees the **bat**.
(2) Target word: “cat”
  a. *Congruent expectancy*: The mice are afraid of the **cat**.
  b. *Conflicting expectancy*: The boy hits the ball with the **cat**.
  c. *Neutral*: The boy sees the **cat**.

Combinations of sentence carriers and final words resulted in a total of 120 sentences with (1) congruent expectancy (*N* = 40), (2) conflicting expectancy (*N* = 40), and (3) neutral expectancy (*N* = 40).

In order to evaluate the strength of these sentences’ expectancy information for predicting the sentence-final word in the congruent condition, an independent sample of 158 adults (mean age = 24.23 years, range = 18–55 years old) completed subsets of sentence carriers with the first word that came to mind (Taylor, 1953). Respondents completed this task via an online questionnaire. The mean proportion of responses completed with the congruent target in the online questionnaire was 81.87 ± 15.1% (mean ±*SD*; see Supplementary Material for stimuli and response proportions). Expectancy information was further controlled in the actual study by using a closed-set task.

The sentence carriers and target words were recorded separately and cross-spliced in order to control for any effects of coarticulation on speech recognition. Sentences were divided into two lists of 60 sentences each (20 sentences for each expectancy condition), such that there was an equal distribution of final word contrasts between each list and so that half the target word pairs occurred only in one list and the other half occurred in the other. Thus, sentences were blocked so each target word in a particular list was presented in sentence carriers of every expectancy condition in that list. This was done because (as described below) each list was presented as a block with a single signal-to-noise ratio (SNR) condition to an individual participant, so this procedure ensured that the SNR manipulation would be across target words for a given participant. Random subsets of these sentences were used to create two lists of 30 sentences each, where each participant would hear one of these lists in quiet (no background noise present).

During the task, sentences were presented in the presence of steady-state speech-shaped noise. This noise was generated in MATLAB and was filtered to match the long-term average spectrum of the sentences. Sentences from the study were concatenated into a single file to generate this noise.

### Procedure

**FIGURE 1:**
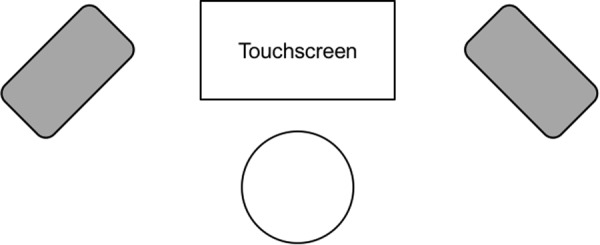
Schematic for the four-alternative forced choice task, where the circle represents the participant’s position in relation to the touchscreen and speakers (represented by the gray boxes). Each speaker is positioned 43 inches away from the participant.

**FIGURE 2:**
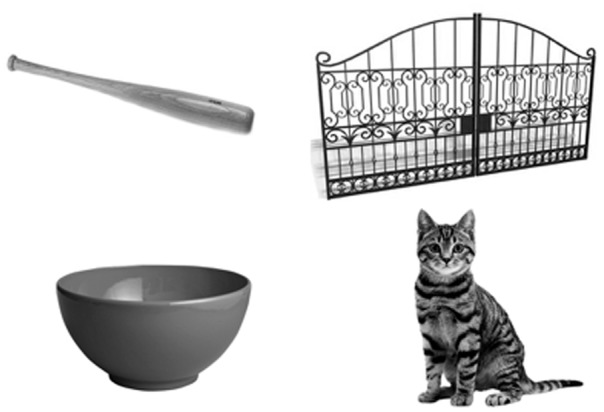
Image arrangement for the four-alternative forced choice task. The images shown correspond to the examples of the expectancy conditions presented earlier. Starting at the top left and proceeding clockwise: the congruent expectancy target (bat), random image (gate), the conflicting expectancy target (cat), and a competitor with the same initial sound as the correct target (bowl).

Participants sat in front of a 21.5″ Dell touchscreen monitor with one Genelec speaker each to the left and right of the monitor (**Figure 1**). Each sentence list was presented in the presence of steady-state speech-shaped noise at either -12 or -7 dB SNR, where the speech-shaped noise remained constant at 62 dB sound pressure level (SPL). List order and SNR condition were counterbalanced across participants. A final block of 30 sentences was presented in quiet. While listening to sentences, participants were presented with four images and completed a four-alternative-forced-choice task where they are asked to identify the image corresponding with the last word of the sentence (Fallon et al., 2002; McMurray et al., 2002). The four images on the screen consisted of: (1) the congruent expectancy target, (2) the conflicting expectancy target, (3) a competitor with the same initial sound as the correct target, and (4) a randomly selected object (**Figure 2**). Images were arranged so that all images appeared the same number of times within the experimental procedure.

### Analysis

Performance on the speech recognition task was quantified by *accuracy* and *RT*. Accuracy was defined as the proportion of correct responses for each expectancy condition in each listening condition. Responses were considered correct if participants chose the image that matched the acoustic target in each sentence, regardless of whether it was semantically aligned with the sentence carrier. RT was defined as the duration of time between the offset of the sentence and the participant’s selection (via touch screen).

## Results

### Accuracy

Participants were tested on their ability to recognize sentence-final words of a spoken sentence by selecting a visual image on a touchscreen. Two trials were removed from analysis because, for these trials, the participants selected an image before the sentence ended. In addition, all trials for one pair of target words (bump/pump, *N* = 6 trials per participant) were excluded from analyses due to a technical error.

**FIGURE 3:**
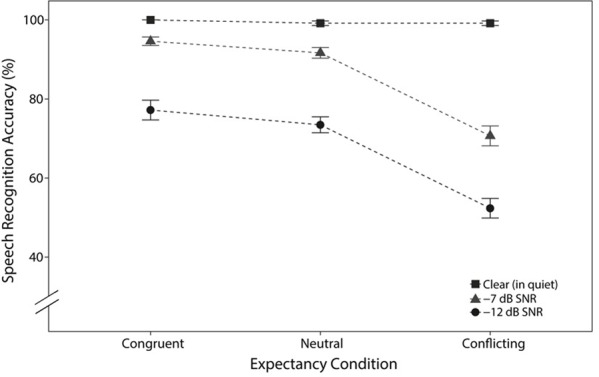
Mean (±SE) for task accuracy, averaged across participants, for each expectancy and listening condition.

Accuracy for each trial in the four-alternative-forced-choice task was binary. Data are visually presented as percent correct ± standard error, which were averaged per participant per expectancy condition (congruent, neutral, conflicting) and listening condition in **Figure 3**. Performance in the clear condition was at ceiling across all expectancy conditions, with a total of four incorrect trials among all of the participants. Because logistic regression becomes unstable for near-perfect performance, trials in the clear condition were excluded from analyses.

Data were subjected to a logistic mixed-effects model to determine the effects of expectancy condition and listening condition on task accuracy. The models were built using the *lme4* package (Bates et al., 2015) in the R environment (R Core Team, 2016). The fixed effects of listening condition (-7 dB SNR, -12 dB SNR) and expectancy condition (congruent, neutral, conflicting) and their interactions were contrast-coded. Contrasts were defined by successive differences for each effect. Specifically, for the fixed effect of listening condition, we tested whether accuracy changed between -7 dB SNR and -12 dB SNR. For the fixed effect of expectancy condition, we tested whether accuracy changed between the congruent and neutral conditions and between the neutral and conflicting conditions. The model included random intercepts for both participant and target word and maximal random slopes: random slopes of all fixed effects for both participants and target word. *P*-values for fixed effects were obtained by likelihood ratio tests.

The model indicated a significant main effect of listening condition, where task accuracy in -12 dB SNR was significantly worse than that in -7 dB SNR (β = -0.93 logits, *SE* = 0.13, *p* < 0.0001). These data are consistent with prior work showing that accuracy decreases with declining SNR (e.g., Miller et al., 1951). There were also significant main effects of expectancy condition on task accuracy. In particular, as both theories predicted, speech recognition performance for the congruent condition was significantly better than in the neutral condition (β = 0.3 logits, *SE* = 0.15, *p* < 0.05). Additionally, as only the belief shift theory predicted, speech recognition in the presence of conflicting expectancy was significantly worse than that in neutral expectancy (β = -0.8 logits, *SE* = 0.09, *p* < 0.0001). This decline in task accuracy for the conflicting condition was not compatible with the facilitation-only theory, but rather suggests that participants’ beliefs were shifted away from the correct target by the semantic information in the sentence carrier.

Relevant to the secondary goal of this experiment, we tested whether there were any interactions between semantic expectancy and listening condition, and there were none. This result of additivity of semantic expectancy and target word acoustics in log-odds is consistent with the predictions of optimal integrator or ideal observer theories of speech perception (Bicknell et al., 2016; Bicknell et al., unpublished). There was, however, one marginal interaction term between the contrasts comparing -12 dB SNR to -7 dB SNR and conflicting to neutral (β = 0.18 logits, *SE* = 0.1, *p* = 0.07), suggesting that semantic expectancy had a smaller effect in log-odds on task performance at -12 dB SNR than at -7 dB SNR, or in other words, when the SNR was poorer. If this particular marginal interaction reflects a true underlying difference, its most likely explanation would still not disconfirm the prediction that the size of the effect of semantic expectancy is constant in log-odds. Instead, this would be most naturally explained as indicating that participants had less access to the semantic information of the sentence carrier in the -12 dB SNR condition. That is, because our experiment added noise to the sentence carriers as well as the target words, seeing a *reduced* effect of semantic expectancy in the most difficult SNR would most naturally be explained by participants’ inability to access the acoustic information about the carrier to form a semantic expectancy.

### Reaction Times

**FIGURE 4:**
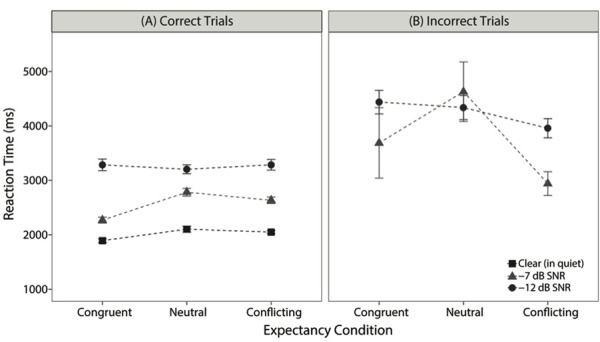
Mean (±SE) for reaction time (RT), averaged across participants, for each expectancy and listening condition. **(A)** RT for correct trials only and **(B)** RT values for incorrect trials only.

Although these two theories of semantic expectancy do not make strong predictions about RTs without additional assumptions, we also analyzed the participants’ RTs to gain a fuller understanding of the results. RT was recorded as the duration (in ms) between the offset of the sentence and the participant’s selection for each trial and binned based on whether participants provided a correct or incorrect selection, as illustrated in **Figure 4**. RT is represented by mean ± standard error for each expectancy condition (congruent, neutral, conflicting) and each listening condition. We consider RT for correct trials here.

Using linear mixed-effects models, we tested for effects of listening condition (clear, -7 dB SNR, -12 dB SNR) and expectancy condition (congruent, neutral, conflicting) on participants’ RT for correct trials while including maximal random effects associated with participants and target word. As in the models for task accuracy described previously, successive difference contrasts were used to evaluate the fixed effects of listening condition (clear, -7 dB SNR, -12 dB SNR) and expectancy condition. *P*-values on fixed effects were again obtained by likelihood ratio tests.

The linear mixed-effects model revealed a significant main effect of listening condition: RT for trials in -7 dB SNR was slower than RT for trials in quiet (β = 309 ms, *SE* = 47, *p* < 0.0001), and similarly, RT for trials in -12 dB SNR was slower than RT for trials in -7 dB SNR (β = 341 ms, *SE* = 57, *p* < 0.0001). When looking at differences in RT across the expectancy conditions, RT for trials with congruent expectancy was significantly faster than RT for trials with neutral expectancy (β = 110 ms, *SE* = 348, *p* < 0.5). In contrast, RTs for trials with conflicting expectancy (i.e., RTs for trials where participants chose the acoustic target) were not significantly different compared to RTs for trials with neutral expectancy (β = -22 ms, *SE* = 31, *p* = 0.45). There was a significant interaction term comparing the contrasts between -12 dB SNR to -7 dB SNR and congruent to neutral (β = -123 ms, *SE* = 42, *p* < 0.01). This interaction indicates that RTs for correct trials were more similar between congruent and neutral expectancy for the -12 dB SNR condition than for the -7 dB SNR condition. Overall, these results suggest that congruent expectancy, the one condition in which the word predicted by the semantic expectancy did in fact appear, promoted faster RTs, and this effect was moderated when the sentence frame was embedded in such a high level of noise that it could not be used to generate a strong expectancy. These results replicate prior studies’ RT results that support the facilitation-only theory, as congruent expectancy decreased RT and conflicting expectancy did not change RT compared to the neutral condition.

### Error Analyses

The above accuracy analysis indicated that, as predicted by the belief shift theory, participants’ beliefs are shifted away from the correct word by conflicting semantic expectancy when processing speech that has been degraded by background noise. This was shown by a lower accuracy in the conflicting condition. But the prediction of the belief shift theory was actually more specific: in the conflicting condition, participants should not just be less likely to choose the correct word (lower accuracy), they should be more likely to choose the word that would have been predicted by the semantic expectancy. Here, we test this further prediction of the belief shift theory by analyzing the responses in the trials in which a participant was incorrect.

**FIGURE 5:**
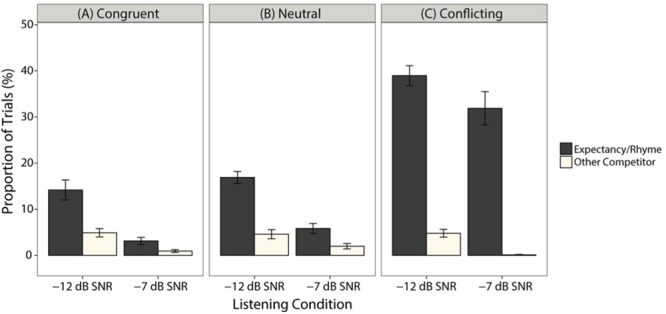
The mean (±SE) proportion of each error type out of total number of trials for each listening condition, averaged across subjects. **(A–C)** shows proportion of each error type for the congruent, neutral, and conflicting expectancy conditions, respectively.

In order to discern whether there was a pattern of errors in each listening condition and each expectancy condition, we analyzed the types of errors made in each listening condition and expectancy condition (**Figure 5**). For these analyses, we focused on errors in the -7 dB SNR and -12 dB SNR conditions because of the small number of errors (i.e., four errors) in the quiet condition. Error rates were calculated out of the total number of trials (i.e., 20 trials for each expectancy condition in each listening condition). Recall that the task was designed so that participants chose among four images: the image that matched the acoustic target and three competitor images (**Figure 2**). The competitor images were classified as either one of the following:

(1) Expectancy competitor, which was the semantically correct word in the conflicting expectancy condition.
(2) Rhyme competitor, which differed from the semantically and acoustically correct target in the congruent expectancy condition by the initial phoneme. In the conflicting expectancy condition, the expectancy and rhyme competitors were the same word.
(3) Initial phoneme competitor, which shared the same initial phoneme as the target.
(4) Random competitor, which is a target for another sentence carrier that is presented in the task.

Accuracy was poorest and, therefore, the number of errors to evaluate were highest in the conflicting expectancy condition. Inspection of the errors in the conflicting expectancy condition (**Figure 5C**) in -12 dB SNR revealed that the majority of errors were due to selecting the expectancy competitor instead of the target (averaged across subjects: 38.95 ± 2.16%). In contrast, selecting another competitor (4.80 ± 0.84%) was not nearly as common. There was a similar pattern in -7 dB SNR, where selecting the expectancy competitor in lieu of the target made up the largest proportion of trials (31.87 ± 3.60%), compared to other competitor types (0.01 ± 0.01%). Evidently, in the conflicting condition, a majority of errors are due to participants selecting the expectancy competitor instead of the target, which supports the predictions of the belief shift theory.

Inspection of the neutral and congruent conditions (**Figures 5B** and **A**, respectively) revealed fewer errors and more distributed competitor selections compared to the conflicting condition. For the neutral condition in -12 dB SNR, there was a larger proportion of selections for the rhyme competitor (16.87 ± 1.3%) than other competitors (4.58 ± 0.97%). This was similar in the congruent condition (rhyme: 14.16 ± 2.16%; other competitors: 4.89 ± 0.9%). In -7 dB SNR, there was a similar pattern in the neutral condition (rhyme: 5.83 ± 1.07%; other competitors: 1.97 ± 0.59%) and congruent condition (rhyme: 3.12 ± 0.79%; other competitors: 0.94 ± 0.28%). Overall, error rates were smaller in the neutral and congruent conditions compared to the conflicting condition, and the rhyme condition was the largest error type here.

Given that the most common error types in the neutral and congruent conditions were rhyme errors, and also that the expectancy competitor in the conflicting condition rhymed with the correct word, we now need to test whether the expectancy error rate in the conflicting condition is significantly higher than the rhyme error rate in the congruent and neutral conditions. If it was not, then instead of being evidence in favor of the belief shift theory, the high rate of expectancy competitor errors in the conflicting condition could be attributed simply to the fact that the expectancy competitor also rhymed with the correct word. Therefore, we performed a statistical analysis of rhyme error rates across conditions with a logistic mixed-effects model. For this analysis of rhyme error rates, each trial was categorized as either a trial on which a rhyme error occurred (including the rhyme competitor in the congruent and neutral condition and the expectancy competitor in the conflicting condition) or a trial on which a rhyme error didn’t occur (including both trials without errors and any other error trial).

For the fixed effect of listening condition, contrasts were coded to test differences between -12 dB SNR and -7 dB SNR. For this analysis, expectancy condition was parameterized differently, with contrast coding comparing the differences between the conflicting and neutral conditions and between the conflicting and congruent conditions. As above, the model included maximal random effects and *p*-values for fixed effects were obtained by likelihood ratio tests.

Consistent with prior analyses, the model indicated a significant main effect of listening condition, where there were more rhyme or expectancy errors in -12 dB SNR compared to -7 dB SNR (β = 0.7 logits, *SE* = 0.13, *p* < 0.0001). Crucially, the model indicated that the conflicting condition had significantly more expectancy selections compared to rhyme selections in the congruent condition (β = -1.25 logits, *SE* = 0.14, *p* < 0.0001) or neutral (β = 1.02, *SE* = 0.12, *p* < 0.0001), confirming the prediction of the belief shift theory. A significant interaction suggested that the difference between the amount of expectancy selections in the conflicting condition compared to rhyme selections in neutral is smaller in -12 dB SNR compared to -7 dB SNR (β = -0.29, *SE* = 0.1, *p* < 0.01). A second significant interaction indicated that the difference between the quantity of rhyme selections in the congruent condition and expectancy selections in the conflicting condition is larger in -12 dB SNR compared to -7 dB SNR (β = 0.31, *SE* = 0.13, *p* < 0.05). Taken together, these results demonstrate that there were more errors due to selecting expectancy competitors in the conflicting expectancy condition compared to rhyme competitor errors in the congruent and neutral condition, confirming the prediction of the belief shift theory.

## Discussion

The primary goal of the present study was to tease apart two theories of how semantic expectancy affects speech perception, which could not be distinguished based on prior work. In the belief shift theory, semantic expectancy was predicted to increase accuracy (relative to a neutral baseline) when the target word is congruent with that expectancy and decrease accuracy when the target word is conflicting with that expectancy. In the facilitation-only theory, semantic expectancy was also predicted to increase accuracy when the target is congruent with the expectancy, but not to affect accuracy when the target is conflicting. Multiple aspects of the results confirmed the predictions of the belief shift theory. The conflicting condition showed significantly and substantially reduced accuracy relative to a neutral baseline in both of the noise conditions, confirming a shift of beliefs away from the correct word. Additionally, an analysis of the errors indicated that this was primarily due to an increased probability of listeners choosing the word that would have been predicted by the semantic expectancy, confirming that this belief shift *away* from the correct word was primarily driven by a shift in beliefs *toward* the word that would have been predicted by the semantic expectancy.

The secondary goal of this study was to test a prediction from ideal observer or optimal integrator models of speech perception that semantic expectancy should combine with acoustic information additively in log-odds space (Bicknell et al., 2016; Bicknell et al., unpublished). As described in the Introduction, additive combination of expectancy and acoustic information in log-odds *qualitatively* predicts that semantic expectancy should have the largest effect on proportion correct (accuracy) when the acoustics are especially ambiguous. This is exactly what we found here. When the sentences were presented in quiet, semantic expectancy had little to no detectable effect on accuracy. When noise was overlaid on the target sentences, participants had lower quality bottom-up information (Rönnberg et al., 2013), rendering the stimuli more ambiguous, and thus semantic expectancy had a larger effect. The present study utilized a -7 dB SNR, where acoustic information is still readily available as participants performed well in both the neutral and congruent conditions (conflicting: 70.67 ± 2.5%; neutral: 91.67 ± 1.3%; congruent 94.61 ± 1.0%) and a -12 dB SNR, which is substantially more difficult (conflicting: 52.36 ± 2.47%; neutral 73.46% ± 2.0; congruent: 77.19 ± 2.5%). The *quantitative* analyses confirmed that this qualitative interaction between semantic expectancy and noise level is exactly as would be predicted by an ideal observer model: there were generally no interactions between semantic expectancy and SNR condition when analyzed with logistic regression. The one exception to this is that semantic expectancy had a marginally *smaller* effect in log-odds for the -12 dB SNR condition, which we interpret as listeners having less access to the semantic context in order to form a semantic expectancy (since the context was also presented at -12 dB SNR).

The results also support the idea that listeners alter their strategies during online language processing: they rely on the bottom-up sensory input, even in the presence of an implausible sentence, when the acoustics are high fidelity, and they engage their semantic knowledge when the acoustics are degraded (Fallon et al., 2002; Pichora-Fuller, 2008). However, the present study’s results provide insight into how exactly semantic expectancy is helpful. It confirms that while semantic expectancy information will improve identification of words when congruent, it can also hurt identification of words in the rare cases where the actual word being said is a close phonological neighbor of a word that would have been predicted from the semantic expectancy. Previous work has shown that acoustic degradation can result in an inability to map acoustic–phonetic features to lexical representations. Additionally, it can also result in perceptual interference where the acoustic–phonetic features are generally obstructed for the listener (Mattys et al., 2012). Both of these consequences of adverse listening conditions could result in semantic expectancy being especially useful as a belief shift, because it would help fill in information missing as a consequence of degradation. Additionally, semantic expectancy provides higher level information that can facilitate mapping acoustic–phonetic features to lexical representations (cf. Rönnberg et al., 2013). Overall, this work clarifies how semantic expectancy works in the presence of acoustic degradation.

The results from the present study raise questions about how semantic expectancy is used in chronic degradation. When an individual has a hearing loss that requires an assistive device (e.g., hearing aid or cochlear implant), the input they receive is consistently degraded. As a result, they may have slower processing times and more lexical uncertainty (McMurray et al., 2016). Smiljanic and Sladen (2013) showed that children with hearing loss benefited from congruent expectancy; however, children with normal hearing still demonstrated more benefit. While the present study was performed using participants with no history of hearing loss, it is an open question whether exposure to chronic degradation, such as from chronic hearing loss, may result in different quantitative patterns to how semantic expectancy and noisy acoustics are integrated. For example, individuals with hearing loss may show greater effects of semantic expectancy in the quiet condition because their experience has led to a shift in their weighting of acoustic vs. semantic information.

Another possible future direction of this work is that the setup of the current study could lend itself to the visual world eye tracking paradigm (Cooper, 1974; Tanenhaus et al., 1995), which tracks looks to each of a set of on-screen objects in real-time as a sentence unfolds. Such a paradigm could provide insight into the time course of how semantic expectancy and acoustics are being utilized and combined as a sentence unfolds.

## Summary

The present study is the first to distinguish two theories of how semantic expectancy affects identification of subsequent words using a speech in noise paradigm. The results supported the belief shift theory predicted by ideal observer models of speech perception (among others), which suggests that semantic expectancy works by biasing responses toward likely words and away from unlikely words. Additionally, the fact that there were no significant interactions of semantic expectancy and noise level supports the further prediction of ideal observer models of speech perception that this effect of semantic expectancy should be of a constant size across levels of noise when analyzed in log-odds. Taken together, these results are consistent with ideal observer models of speech perception, and provide insight into how listeners combine all available information sources to understand what is being said when confronted with poor acoustic input.

## Ethics Statement

This study was carried out in accordance with the recommendations of the Institutional Review Board of Northwestern University with written informed consent from all subjects. All subjects gave written informed consent in accordance with procedures approved by the Institutional Review Board of Northwestern University.

## Author Contributions

KS, KB, and TG-C designed the research and wrote the paper. KS performed the research. KS and KB analyzed the data.

## Conflict of Interest Statement

The authors declare that the research was conducted in the absence of any commercial or financial relationships that could be construed as a potential conflict of interest.
