# Supplementary material for: Belief Shift or Only Facilitation: How Semantic Expectancy Affects Processing of Speech Degraded by Background Noise
Source: Front Psychol. 2018 Feb 8;9:116. doi: 10.3389/fpsyg.2018.00116 (PMC5809983; doi:10.3389/fpsyg.2018.00116)
Supplement: Supplementary file 1 [file Data_Sheet_1.pdf]

Appendix

Each sentence carrier with both the corresponding congruent target (listed first) and conflicting target (listed last). In the final column, we list the proportion of respondents that completed the sentence carrier with the congruent target in the online sentence completion questionnaire.

Sentence	Target Word	Proportion of Responses
The horses wait for the opening of the	gate (congruent)<br>bait (conflicting)	72.80%
The fish takes the hook's	bait<br>gate	81.35%
The boy eats ice cream from a	cone<br>bone	64.75%
The dog chews on a	bone<br>cone	71.55%
The mice are afraid of the	cat<br>bat	72.03%
The boy hits the ball with the	bat<br>cat	91.67%
The boy builds a tower with his	blocks<br>clocks	47.55%
To check the time, look at the	clock<br>block	82.50%
The dog drinks water from the	bowl<br>coal	83.66%
Santa gives bad children a sack of	coal<br>bowl	98.59%
The teenager is excited to drive the	car<br>bar	88.13%
The man orders a drink at the	bar<br>car	93.80%
A fruit with fuzzy skin is a	peach<br>beach	88.49%
The boy made a sandcastle at the	beach<br>peach	96.68%
After falling down, his head had a	bump<br>pump	55.96%

The man inflates the tire with a	pump<br>bump	90.72%
After dinner, the man paid his	bill<br>pill	72.80%
To control cholesterol, the woman took a	pill<br>bill	96.39%
The man put gas in the car's	tank<br>bank	94.16%
The boy put his money in the	bank<br>tank	80%
Stewards urged the passenger to take his	seat<br>feet	83.59%
Ballerinas need shoes that fit their	feet<br>seat	91.61%
To cool herself, she turned on the	fan<br>van	81.33%
The florist delivered flowers in his	van<br>fan	52.00%
The boy shuffled a deck of	cards<br>guards	100.00%
The castle entrance was watched by	guards<br>cards	87.05%
She put honey and lemon in her	tea<br>key	95.19%
The man unlocked the door with a	key<br>tea	98.00%
A genie appears if you rub the	lamp<br>ramp	98.30%
The skate park has many	ramp<br>lamp	62.50%
The quarry was filled with	rocks<br>locks	64.76%
The boy secured his bike with a	lock<br>rock	81.41%
Cleaning the yard is easy with a	rake<br>lake	95.10%
The man goes fishing on the	lake<br>rake	47.96%
A suit is incomplete without a	tie<br>pie	93.06%
After picking apples, the girl baked a	pie<br>tie	91.80%

The man raised the ship's	sail<br>veil	57.85%
On her head the bride wore a	veil<br>sail	86.66%
The exit was marked by a large	sign<br>vine	98.70%
The man picks grapes off the	vine<br>sign	84.31%

Neutral Sentence Carriers

Each sentence carrier for the neutral condition with the final word targets presented in the study. In the results of the online sentence completion questionnaire, final word responses for these sentences were extremely distributed and the following sentence carriers yielded at minimum 41 unique possible final words.

**Sentence**	**Final Word**
He read about the	gate<br>bait
He pointed at the	cone<br>bone
She read about the	cat<br>bat
She looked at the	blocks<br>clocks
He pointed at the	bowl<br>coal
He walks to the	car<br>bar
The girl really likes the	peach<br>beach
The boy looks at the	pump<br>bump
They talk about the	bill<br>pill
He reads about the	tank<br>bank

He looked at the	seat<br>feet
He pointed at the	fan<br>van
They talked about the	cards<br>guards
He picked up the	tea<br>key
She looked at the	lamp<br>ramp
He looked at the	lock<br>rock
She read about the	rake<br>lake
She picked up the	tie<br>pie
They talked about the	sail<br>veil
They looked at the	sign<br>vine
